# Longitudinal Study of Selected Bacterial Zoonoses in Small Ruminants in Tana River County, Kenya

**DOI:** 10.3390/microorganisms10081546

**Published:** 2022-07-30

**Authors:** Martin Wainaina, Johanna F. Lindahl, Ian Dohoo, Anne Mayer-Scholl, Kristina Roesel, Deborah Mbotha, Uwe Roesler, Delia Grace, Bernard Bett, Sascha Al Dahouk

**Affiliations:** 1 Department of Biological Safety, German Federal Institute for Risk Assessment, 12277 Berlin, Germany; anne.mayer-scholl@bfr.bund.de (A.M.-S.); sascha.al-dahouk@gmx.de (S.A.D.); 2 Department of Veterinary Medicine, Freie Universität Berlin, 14163 Berlin, Germany; k.roesel@cgiar.org (K.R.); deborahmbotha@gmail.com (D.M.); 3 Animal & Human Health Program, International Livestock Research Institute, Nairobi 00100, Kenya; j.lindahl@cgiar.org (J.F.L.); d.randolph@cgiar.org (D.G.); b.bett@cgiar.org (B.B.); 4 Zoonosis Science Center, Department of Medical Biochemistry and Microbiology, Uppsala University, 75237 Uppsala, Sweden; 5 Department of Clinical Sciences, Swedish University of Agricultural Sciences, 75007 Uppsala, Sweden; 6 Centre for Veterinary Epidemiologic Research, University of Prince Edward Island, Charlottetown, PE C1A 4P3, Canada; dohoo@upei.ca; 7 Institute for Animal Hygiene and Environmental Health, Freie Universität Berlin, 14163 Berlin, Germany; uwe.roesler@fu-berlin.de; 8 Natural Resources Institute, University of Greenwich, Kent ME4 4TB, UK; 9 Department of Internal Medicine, RWTH Aachen University Hospital, 52074 Aachen, Germany

**Keywords:** brucellosis, leptospirosis, Q fever, seroconversion, co-infection, sheep and goats, land-use changes, East Africa

## Abstract

Brucellosis, Q fever, and leptospirosis are priority zoonoses worldwide, yet their epidemiology is understudied, and studies investigating multiple pathogens are scarce. Therefore, we selected 316 small ruminants in irrigated, pastoral, and riverine settings in Tana River County and conducted repeated sampling for animals that were initially seronegative between September 2014 and June 2015. We carried out serological and polymerase chain reaction tests and determined risk factors for exposure. The survey-weighted serological incidence rates were 1.8 (95% confidence intervals [CI]: 1.3–2.5) and 1.3 (95% CI: 0.7–2.3) cases per 100 animal-months at risk for *Leptospira* spp. and *C. burnetii*, respectively. We observed no seroconversions for *Brucella* spp. Animals from the irrigated setting had 6.83 (95% CI: 2.58–18.06, *p*-value = 0.01) higher odds of seropositivity to *C. burnetii* than those from riverine settings. Considerable co-exposure of animals to more than one zoonosis was also observed, with animals exposed to one zoonosis generally having 2.5 times higher odds of exposure to a second zoonosis. The higher incidence of *C. burnetii* and *Leptospira* spp. infections, which are understudied zoonoses in Kenya compared to *Brucella* spp., demonstrate the need for systematic prioritization of animal diseases to enable the appropriate allocation of resources.

## 1. Introduction

Bacterial infections are the leading causes of non-malarial febrile illnesses in Africa [1]. Since a majority of these diseases are of animal origin [2], control measures that consider animal hosts are not only indispensable but also cost-effective [3]. Brucellosis, leptospirosis, and Q fever are priority bacterial zoonoses in Kenya, mainly because of the high disease severity. Despite this, their epidemiology is substantially understudied [4,5,6]. In addition, studies investigating the co-occurrence of infections are scarce.

Brucellosis is caused by bacteria of the genus *Brucella*, and *B. melitensis*, *B. abortus*, and *B. suis* are responsible for most human cases globally [7]. Brucellae have animal host preference, with *B. melitensis* commonly associated with sheep and goats, *B. abortus* with cattle, and *B. suis* with pigs, hare, and caribou [8]. Human brucellosis is a debilitating illness mostly characterized by fever, joint pain, hepato-, and splenomegaly [9]. Human brucellosis is primarily transmitted through direct contact with infected animals or their body fluids or the consumption of raw milk and milk products. Brucellosis in low-income countries is responsible for huge economic losses brought about by reproductive wastage and loss of productivity in domestic animals [10].

Leptospirosis is a bacterial zoonosis caused by bacteria of the genus *Leptospira*. The genus comprises 64 identified genomospecies that are serologically representative of more than 300 serovars [11,12]. Closely related serovars are grouped into serogroups. Leptospires are transmitted through contact with fresh water or soil that is contaminated with urine from infected animals [13]. The bacteria have a predilection for the renal tubes of maintenance hosts such as rodents and domestic animals, which can transfer the infection to other animals and humans (accidental hosts) [13]. Leptospirosis can cause abortions and stillbirths in domestic animals [14]. Human cases often present with fever, accompanied by chills, headaches, muscle pain, and abdominal pain. Severe and fatal outcomes can also occur [13].

Q fever (or coxiellosis in livestock and wildlife) is a zoonotic bacterial infection caused by *Coxiella burnetii*. The bacteria transition from virulent to avirulent states in a phenomenon termed phase variation that comprises phase I, which is associated with infection in mammals, and phase II, which is a less virulent form and defined by deletion or truncation of the lipopolysaccharide virulence factor. Acute infection is mostly characterized by antibody response against phase II antigens and chronic infection against both phase I and II antigens. Transmission to humans usually happens through inhalation of aerosols from contaminated excreta or birth products of infected animals but can also occur through untreated milk. Infection is asymptomatic in most cases but can present with flu-like symptoms when in the mild form, and chronic and fatal illnesses have been documented. Infection in livestock often lacks clear signs, with only a few cases showing sporadic abortions [15]. Ticks may contribute to the transmission of the bacteria from livestock species to humans, but the extent of this is under debate [16].

Sheep and goats are important sources of meat in Kenya and contribute to the livelihoods of many [17,18]. However, seroprevalence and incidence estimates of the previously mentioned bacterial zoonoses in small ruminants are largely unknown. These infections could pose a serious threat to human as well as animal health and cause economic losses, especially affecting low-income populations. This particularly applies to Kenya’s vast and vulnerable arid and semi-arid land (ASAL) areas, which have increasingly undergone changes in land use that enable crop production, such as the revival of irrigation schemes. This is to improve the livelihoods of populations from these areas, as they are part of Kenya’s most resource-scarce areas. However, land-use changes can influence the pattern of vector-borne diseases [19]. Less is known about the effect of these changes on bacterial infections.

Therefore, we investigated *Leptospira* spp., *Brucella* spp., and *C. burnetii* in sheep and goats from a pastoral, irrigated, and riverine ecosystem in Tana River County of Kenya. Specifically, we estimated the survey-weighted seroprevalence and incidence rates of these three priority zoonoses in the three study sites. We also aimed at identifying risk factors for seropositivity.

## 2. Materials and Methods

### 2.1. Study Area

**Figure 1:**
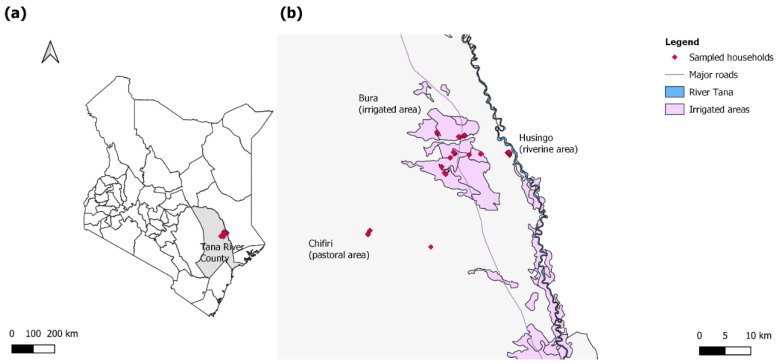
(**a**) A map of Kenya highlighting Tana River County in gray and the study sites in red; (**b**) a map highlighting the households sampled in the three sites (red). The sites included the pastoral Chifiri village, the riverine Husingo village, and the settlements in the Bura irrigation scheme.

Samples for this study were collected from the Bura irrigation scheme, Husingo, and Chifiri villages of Tana River County (Figure 1), an ASAL area in Kenya, using a longitudinal study design as previously described by Mbotha et al. [20]. The Bura irrigation scheme is an irrigation area and Husingo village represents a riverine ecosystem due to its proximity to the river Tana (a major source of water for Tana River County) and its surrounding forests. Chifiri village represents a pastoralist region where mixed grazing of animals is practiced and larger livestock populations are present. The county also has a bimodal rainfall distribution, which consists of short (around October to December) and long rains (around March to May). Sample collection was conducted between September 2014 and June 2015 and included a total of 316 small ruminants (88 sheep and 228 goats) followed up in September, November, and December of 2014, and January, March, and June of 2015. A total of 247 animals were selected for follow-up at the beginning of the study, and 55 were lost to follow-up during the course of the study. These were replaced by 69 animals. A detailed distribution of animals sampled and replaced during each visit is presented by Mbotha et al. [20]. Consequently, the study accrued a total of 1949 animal-months. The selection of the villages in the study sites was purposive (to obtain the three study settings), and selecting households in the villages as well as animals in a household was done randomly from a sampling frame using computer-generated random numbers. Finer details of the study area, selection of households and animals for sampling and follow-up, as well as data collection are also as previously reported by Mbotha et al. [20].

### 2.2. Laboratory Analyses

#### 2.2.1. *Brucella* spp.

The serum samples were screened for antibodies against *Brucella* spp. using an indirect IgG enzyme-linked immunosorbent assay (ELISA) (ID Screen Brucellosis Serum Multi-species, ID-Vet, Grabels, France) and the Rose Bengal test (RBT) (Pourquier^®^ Rose Bengale Ag, IDEXX, Montpellier, France) in parallel according to the manufacturers’ instructions. Results were confirmed using the complement fixation test (CFT) (Institut Virion/Serion GmbH, Würzburg, Germany) as recommended by the World Organisation for Animal Health (WOAH, formerly the OIE). Samples with international CFT units (ICFTU)/mL ≥20 were considered positive. Both RBT and CFT can detect IgG and IgM antibodies. DNA was isolated from the serum of animals that were seropositive at any sampling time point using the DNeasy^®^ Blood and Tissue Kit (Qiagen, Hilden, Germany). We also tested for *Brucella* spp., *B. abortus*, and *B. melitensis* using quantitative polymerase chain reaction (qPCR) targeting the *bcsp31* gene and the IS*711* intergenic element [21,22].

#### 2.2.2. *Leptospira* spp.

Serological screening for leptospires was performed using the microscopic agglutination test (MAT) as recommended by the WOAH. The serovars (serogroup, reference strain) included in the MAT panel were: Icterohaemorrhagiae (Icterohaemorrhagiae, RGA); Ballum (Ballum, Mus 127); Grippotyphosa (Grippotyphosa, Moskva V); Australis (Australis, Ballico); Pomona (Pomona, Pomona); Sejroe (Sejroe, M 84); Canicola (Canicola, Hond Utrecht IV); and Hebdomadis (Hebdomadis, Hebdomadis). These serovars are recommended for testing animals and humans in East Africa [23]. Samples with MAT titers ≥1:100 were regarded as positive. MAT detects both IgG and IgM antibodies and is currently considered the serological gold standard test for leptospires. DNA was extracted from whole blood samples of animals that were seropositive at any sampling time point and tested using qPCR targeting the *lipL32* gene that identifies pathogenic leptospires [24,25].

#### 2.2.3. *C. burnetii*

Serological screening for *C. burnetii* IgG antibodies was performed using the ID Screen^®^ Q fever indirect multi-species ELISA (IDVet, Montpellier, France) as per the manufacturer’s recommendations. The kit uses *C. burnetii* Nine Mile phase I and phase II antigens and is validated for testing cattle, sheep, and goat sera. Samples with a percent sample-to-positive ratio (S/P%) >50 were considered positive, and those with 50 ≥ S/P% > 40 were inconclusive. Samples with S/P% ≤ 40 were considered negative. DNA was extracted from serum samples of animals that were seropositive at any sampling time point and tested using qPCR targeting the IS*1111* element [26].

A summary of the primers and probes used in this study is presented in Table 1.

### 2.3. Data Analyses

Laboratory data were recorded on MS Excel and imported into R statistical software environment version 4.0.0 (R Core Team, Vienna, Austria) [27], where they were merged with animal and household data. Statistical analyses were threefold.

Firstly, we determined seroprevalence estimates using results from the first sampling of each animal. Animals that initially had borderline results but tested positive in later sampling points were regarded as positive from the beginning and included in this first analysis. As exposure to the pathogen in these cases most likely happened before our first time point, we did not consider them as seroconversions in our analyses since we could not estimate their time to seroconversion. They are nonetheless good indicators of disease presence. Animals that tested borderline and were tested only once were excluded from these analyses because we could not confirm their exposure status. The seroprevalence estimates and their confidence intervals were computed with adjustments for the complex survey design by considering both the sampling weights and the multistage nature of the sampling. Total sampling weights were determined by finding the product of the inverse probability of an animal being sampled in a household (also herd) and a household being sampled in a village, as explained by Dohoo et al. [28]. Analysis was performed using the *svydesign* command in the *survey* package version 4.0 [29], and villages were used as the primary sampling units. However, when estimating seroprevalence by site, in sites where only one village was sampled (riverine and pastoral), we used households as the primary sampling units. We also fitted a survey-weighted logistic model of the *Leptospira* spp. results with those of *C. burnetii* and *Brucella* spp. and included the site variable as a potential confounder. We additionally fitted a survey-weighted model of *C. burnetii* with the two other zoonoses and site. This was meant to determine the odds of an animal being seropositive to a second zoonosis when seropositive to an initial zoonosis.

Secondly, we performed risk factor analyses for seroprevalence. Based on the literature [8,14,16], we considered the following putative risk factors: species (sheep or goat); sex (female or male); age (young [kids, lambs] or adult animals); reproductive status (active [breeding, lactating, pregnant] or inactive [not breeding]); site (pastoral [Chifiri]), irrigation [Bura] or riverine [Husingo]); and herd size (three categories based on the distribution of small ruminants in the household at the 50th and 75th percentiles). Before building the logistic regression models, we determined the relationship of these potential risk factors with the outcome variable using a causal diagram (also known as a direct acyclic graph) on the browser-based environment DAGitty^®^ (http://www.dagitty.net, accessed on 21 March 2022). We chose the site variable as the main exposure variable and the serological results as the outcome variable and combined age and sex to simplify the causal diagram. All variables to the right of the main exposure variable were regarded as intervening variables (also intermediary or mediator) and therefore not admitted to the logistic regression models.

**Figure 2:**
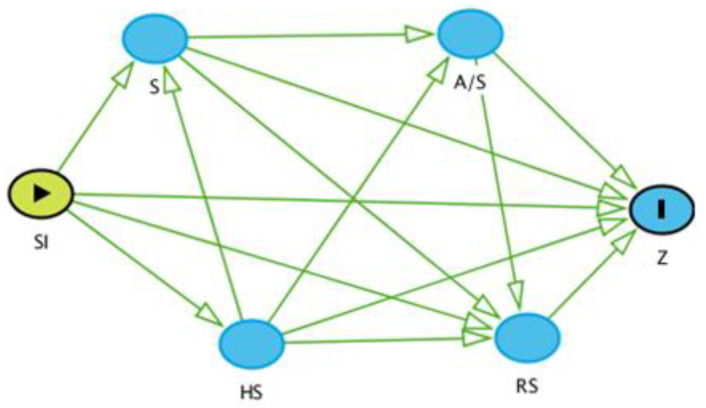
A causal diagram of the potential risk factors with the outcome variable (Z), which is seropositivity. SI: Site; S: Species; A/S: Age/Sex; RS: Reproductive Status; HS: Herd Size.

We took this approach to determine the total effect of our main exposure variable (site) on the outcome variable (seropositivity), which requires the lack of known intervening variable(s) between the exposure of interest and disease outcome. Including intervening variables when controlling for confounding may produce spurious causal effects by biasing the measure of association. This is because part of the effect is removed by the intervening variable(s) [28]. As all variables in our metadata were intervening (Figure 2), we only performed univariable analyses of the site variable when determining total effects. In addition, we determined the direct effects of the site variable. This initially involved survey-weighted univariable logistic regression models of the variables of interest. All variables that were significant (*p*-value < 0.1) were admitted to the multivariable model. We started with the full model, which contained all the variables significant at the univariable level, and removed them using a backward elimination approach until there was no evidence of confounding effects. The survey-weighted logistic regression models were fitted using the *svyglm* command and considered inverse probability weighting and design-based standard errors as previously detailed.

Thirdly, we analyzed the seroconversion data. Seroconversions were defined as animals that were seronegative at the time of first sampling but later seropositive during our study. We initially determined the distribution of seroconversions within sites. As the number of seroconversions was few per primary sampling unit, we neither estimated weighted proportions nor performed risk factor analyses for seroconversions. However, we computed the serological incidence rates. For these analyses, seroconversions were regarded as disease cases, even though the animals may not have necessarily exhibited clinical manifestations. The animal-months at risk were determined by considering the time difference between the first sampling of animals and midway between the last negative and first positive test result. Animals that did not seroconvert contributed the entire period they were followed up. Animals that were only sampled once were not considered for these analyses since they did not have enough time relative to the other animals to seroconvert. We fitted survey-weighted Poisson regression models of the seroconversion variable and used log-transformed animal-months at risk as the offset for *Leptospira* spp. and *C. burnetii*. The estimates were done using the *svyglm* function, and the *subset* argument was used to obtain separate estimates for sheep and goats. Summary statistics for the models were obtained using the *broom* package version 0.7.6 [30]. We lastly plotted the seroconversions observed for both bacteria against mean monthly maximum and minimum temperatures and rainfall received in the area to examine any obvious distribution patterns.

## 3. Results

### 3.1. Study Overview

We selected 228 (72.2%) goats and 88 (27.9%) sheep from 37 households in 8 villages in this study. The median number of adult people (>18 years) in households in the study area was 4 (interquartile range [IQR]: 2–5) while that of children <10 years and those 10–18 years was 3 (IQR: 1–4) and 2 (IQR: 1–3), respectively. There were more animals selected from the irrigated settings (*n* = 139, 44.0%) than riverine (*n* = 108, 34.2%) or pastoral settings (*n* = 69, 21.8%). The villages in the irrigated area had the following number of selected animals: 55 in Village Five, 28 in Village Seven, 25 in Village Two, 13 in both Village Six and Nine, and 5 in Village Eight. The riverine Husingo and pastoral Chifiri villages had 108 and 69 animals selected, respectively.

### 3.2. Seroprevalence Estimates

The overall weighted seroprevalence estimate for *C. burnetii* was highest at 34.6% (95% confidence intervals [CI]: 24.3–47.0). Villages in the irrigated setting had the following distribution of seropositive animals: 5/5 (100%) in Village Eight, 6/13 (46.2%) in Village Nine, 16/55 (29.1%) in Village Five, 7/28 (25%) in Village Seven, 4/25 (16%) in Village Two, and 1/13 (7.7%) in Village Six. The pastoral Chifiri and riverine Husingo villages had 25/69 (36.2%) and 7/108 (6.5%), respectively. The pastoral region had the highest seroprevalence estimates, followed by those of the irrigated and riverine areas.

The overall weighted seroprevalence estimate for *Leptospira* spp. was the second highest in our study at 15.3% (95% CI: 11.6–20.0). The irrigated area had the following number of seropositive animals: 4/13 (30.8%) in Village Nine, 3/13 (23.1%) in Village Six, 1/5 (20%) in Village Eight, 4/25 (16%) in Village Two, 3/54 (5.6%) in Village Five, and 0/28 in Village Seven. The riverine Husingo village had the highest seroprevalence in the study (26.9%, 95% CI: 15.5–42.0) and the pastoral Chifiri village had the least (13.9, 95% CI: 5.3–32.0).

The lowest overall was for *Brucella* spp., and due to the few seropositive animals (4/316, 1.3%), we could not estimate weighted seroprevalence reliably. We also observed anti-complementary activity in two samples tested using the CFT. However, these samples were from two seronegative animals, which also did not seroconvert.

The distribution of the proportion of seropositive animals for all three zoonoses with the various variables is presented in Table 2, and weighted seroprevalence estimates and standard errors for the bacterial pathogens with sites are in Table 3. The somewhat wide confidence intervals for the weighted seroprevalence estimates demonstrate that our study may have been statistically underpowered.

### 3.3. Co-Exposures

We observed one animal exposed to both *Brucella* spp. and *Leptospira* spp., and two to *Brucella* spp. and *C. burnetii*. Twelve animals were exposed to both *Leptospira* spp. and *C. burnetii*, and we observed no animals co-exposed to all three pathogens of interest. Results of the survey-weighted logistic regression models for co-exposure showed that animals that were *Leptospira*-positive had 2.51 (95% CI: 2.28–2.77, *p*-value < 0.01) times higher odds of being seropositive to *C. burnetii*. In addition, animals seropositive to *C. burnetii* had 2.52 (95% CI: 2.29–2.77, *p*-value < 0.01) higher odds of being *Leptospira*-seropositive than those that were seronegative.

### 3.4. Seroconversions

We observed 10 seroconversions to *C. burnetii* in the 218 animals considered for follow-up (Table 2), with 7 being from the irrigated area and 3 from the pastoral area. There were no seroconversions in the riverine area. We found the following number of seroconversions in villages in the irrigated study area: 5/21 (23.8%) in Village Two, 1/7 (14.3%) in Village Nine, and 1/37 (2.7%) in Village Five. Village Six, Seven, and Eight had 0/12, 0/18, and 0/5 seroconversions, respectively. The pastoral Chifiri and riverine Husingo villages had 3/30 (10.0%) and 0/88 seroconversions, respectively.

We observed the highest seroconversions to *Leptospira* spp. (*n* = 27) in 226 animals followed up (Table 2). There were more seroconversions in the riverine area (13) than in the irrigated (10) and pastoral areas (4). Villages in the irrigated area had the following number of seroconversions: 2/10 (20%) in Village Six, 4/21 (19.0%) in Village Two, and 3/48 (6.3%) in Village Five. Village Seven and Eight had 0/24 and 0/4 seroconversions, respectively. We observed 13/68 (19.1%) and 4/44 (9.1%) in the riverine Husingo and pastoral Chifiri villages, respectively.

There were 267 animals followed up for the *Brucella* spp. study, but we observed no seroconversions (Table 2).

We found co-occurrence of seroconversions to both *Leptospira* spp. and *C. burnetii* in five animals. Of these, three were from the irrigated area and two from the pastoral area.

**Figure 3:**
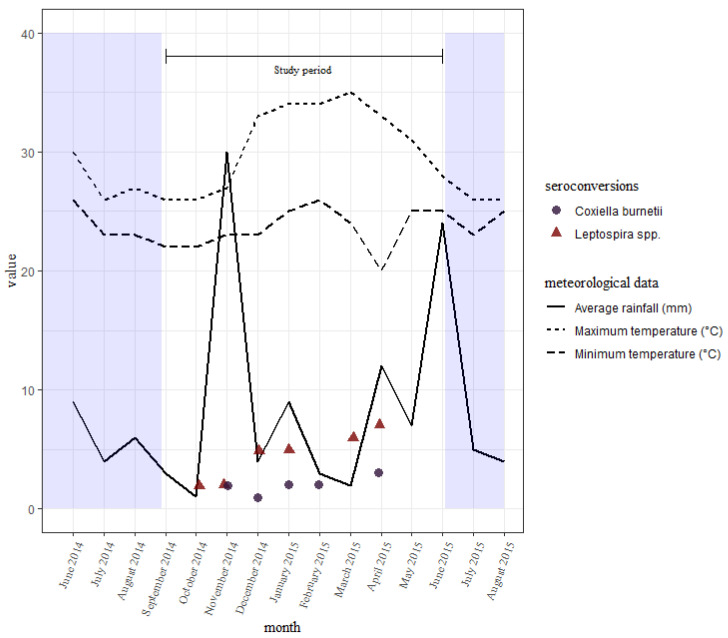
A distribution of seroconversions to *Leptospira* spp. and *C. burnetii* over time with the average rainfall received and temperatures reported every month in the study area. Sampling was done in September, November, and December of 2014, and in January, March, and June of 2015.

The seroconversions for *C. burnetii* occurred all through the study period, with no clear differences between the wet and dry seasons. Those of leptospires were also all through the study period, with slightly fewer being seen earlier on during the short rains than the long rains towards the end (Figure 3). There were slightly more seroconversions during the wet season than the dry.

### 3.5. Serological Incidence Rates

We estimated the serological incidence rates from these seroconversions and expressed them as the number of cases per 100 animal-months at risk. The serological incidence rate estimates were the highest for leptospires at 1.8 cases per 100 animal-months at risk (95% CI: 1.3–2.5 overall, 95% CI: 1.4–2.4 in sheep, and 95% CI: 1.2–2.6 in goats). There were lower estimated serological incidence rates of 1.3 cases per 100 animal-months at risk for *C. burnetii* in the study (95% CI: 0.7–2.3 overall, 95% CI: 0.6–3.1 in sheep, 95% CI: 0.7–2.3 in goats). We did not observe any seroconversions for *Brucella* spp., resulting in a low estimate of 0.0 cases per 100 animal-months at risk.

### 3.6. Risk Factor Analyses

We performed univariable logistic models with seroprevalence as the outcome variable and site as the main exposure variable for both the *C. burnetii* and *Leptospira* spp. data and excluded *Brucella* spp., as well as all data on seroconversions due to few data points per primary sampling unit. Results of the logistic models with considerations for complex survey designs are given in Table 4.

For *C. burnetii*, when compared to the animals in the riverine setting, those in the irrigated and pastoral areas had odds of seroprevalence that were 6.8 (95% CI: 2.1–22.1) and 13.6 (95% CI not available due to only one village in each of pastoral and riverine regions) times higher, respectively. There was no significant difference between the irrigated and pastoral settings. The direct effects of the site variable, as determined by the final survey-weighted multivariable logistic regression, were largely similar to the total effects (Table 5). This was except for the irrigated category that showed lowered direct effects, a result likely from the influence of intervening variables added to the model. All the variables were significant from the univariable analyses (*p*-value < 0.1) that preceded these final models. The study site was not significantly associated with seroprevalence of *Leptospira* spp. (Table 4 and Table 5), although the lowest seroprevalence was observed in pastoral areas and the highest in the riverine area.

### 3.7. Real-Time PCR Testing

We retested all the samples of the four animals that were serologically positive at any sampling time point using qPCR for *Brucella* spp., *B. abortus*, and *B. melitensis*. The first animal (No. 48) was an adult female goat from the irrigated area. The goat was also from a mid-sized herd (13–35 animals) and was consistently PCR-positive from the second to the last sampling while seropositive on the first sampling time point only. Quantification cycle (Cq) values were generally high, suggesting low bacterial load. The second animal (No. 102) was a young male goat from a large herd (>35) in the irrigated area. The animal was sampled only once and was both PCR- and serologically positive. The third animal (No. 115) was an adult female goat from the irrigated area. The animal was seropositive only on the first sampling time point and tested PCR-positive intermittently for the rest of the study period. The animal was from a small herd (<13). The last animal (No. 370) was an adult female sheep from the riverine area and was from a mid-sized herd (13–35 animals). The animal was intermittently PCR-positive between the first and last sampling points. However, it was consistently seropositive for the entire study period. All four animals were reproductively active. A summary of the timelines and the laboratory results are presented in Table 6.

**Figure 4:**
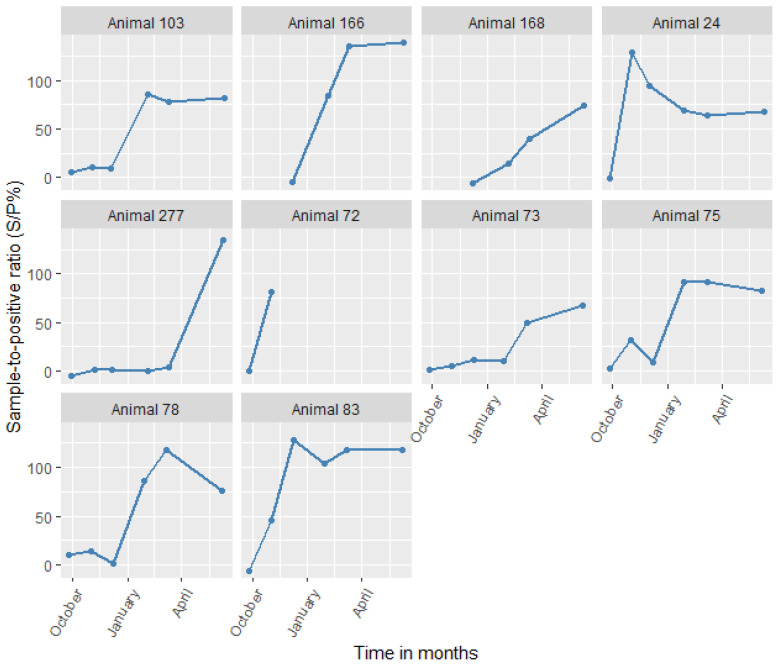
Changes in antibody levels for all animals that seroconverted to *Coxiella burnetii* (in S/P%) with time.

We also screened animals that were positive for *C. burnetii* by serology at any sampling time points using PCR. Despite animals showing high antibody titers for sustained periods (Figure 4), only one animal (No. 75) tested positive by PCR in the study. The animal was an adult female sheep from the irrigated area that was reproductively active and from a large herd (>35 animals). The animal tested PCR-positive only on the third sampling (December 2014). Subsequently, it was seropositive from the fourth (January 2015) to the last sampling (June 2015), essentially seroconverting. The animal had a high Cq value (38), suggesting that there was a low bacterial load.

We did not detect any leptospires by PCR. Additionally, we observed no co-occurring pathogens by PCR (i.e., at least two of the three target bacteria).

### 3.8. Leptospiral Serovars

We found 48 seropositive animals at the first sampling time points. Of these, 31 agglutinated with serovar Ballum, making it the most prevalent serovar in the study area. Other agglutinating serovars were: Sejroe/Ballum (5), Australis (5), Pomona/Ballum (2), Sejroe (1), Pomona/Sejroe/Ballum (1), Australis/Grippotyphosa (1), Icterohaemorrhagiae (1), and Grippotyphosa (1). Goats had the following agglutinating serovars: Ballum (28), Australis (5), Sejroe/Ballum (4), Pomona/Ballum (2), Sejroe (1), Pomona/Sejroe/Ballum (1), Australis/Grippotyphosa (1), Icterohaemorrhagiae (1), and Grippotyphosa (1). We identified Ballum (3) and Sejroe/Ballum (1) in sheep.

When we compared serovars at the first and last sampling time points, we found 11 animals with varying serovars. These variations between the first and last results comprised: Sejroe/Ballum to Ballum (6), Sejroe/Ballum to Sejroe (1), Australis/Pomona to Ballum/Pomona (1), Sejroe to Ballum (1), Pomona to Ballum (1), and Ballum to Pomona (1).

## 4. Discussion

We sampled 316 small ruminants in Tana River County and followed up those that were seronegative at the first sampling for nine months. The serological incidence rates for *Leptospira* spp. and *C. burnetii* were 1.8 and 1.3 cases per 100 animal-months at risk, respectively. We also determined that animals in irrigated and pastoral settings had 6.83- and 13.61-times higher odds of being seropositive to *C. burnetii* than those from the riverine area. Lastly, we observed multiple co-exposures to *C. burnetii* and *Leptospira* spp., and animals exposed to one zoonosis had at least 2.5 times higher odds of exposure to a second zoonosis. The high prevalence of understudied zoonoses (*Leptospira* spp. and *C. burnetii*) and relatively lower prevalence of the more studied one (*Brucella* spp.) highlight the need for systematic disease prioritization.

### 4.1. Seroprevalence, Seroconversions, and Risk Factors

Comparable seroprevalence estimates of *C. burnetii* have been observed in ASAL areas in Kenya, with slightly higher estimates in goats than sheep [5,31,32,33]. We found higher weighted estimates in the pastoral settings than in both the irrigated and riverine settings. These pastoral settings are characterized by close contact between animals, especially through communal grazing and common watering points. Transmission of the bacteria in the large herds through contaminated dust particles is therefore likely. It is also likely that transmission often happens in animals that are breeding, pregnant, or lactating in the herds, especially since the communities under study lack adequate extension services that would provide access to artificial insemination. Breeding in tropical countries also lacks the seasonality brought about by annual seasons, making perennial transmission via this route possible. We also observed animals from irrigated settings having higher odds of seropositivity than those from riverine settings. Irrigation could be a risk factor for transmission of *C. burnetii* when animal waste is used as fertilizer; animal waste seeps into irrigation canals, contaminated soils are aerosolized, and rodents disperse *C. burnetii* in the environment [34]. Measures that can control coxiellosis include vaccination of herds, controlling environmental transmission (e.g., by using properly composted manure, controlling rodents, separating periparturient animals, burying aborted materials), and quarantine [35]. In addition, raising community awareness on proper biosecurity measures may help lower human exposure.

Few studies have estimated the exposure of sheep and goats to leptospires in Kenya, and similarly high seroprevalence estimates have been found in other ASAL regions [36,37,38]. We observed the highest seroprevalence in animals in the riverine ecosystem. This was further corroborated by the most seroconversions to leptospires being in this area. Infected animals shed the leptospires via urine, which leads to immense contamination of soil and water. The bacteria can survive in soil from riverbanks for up to nine weeks, and the risk may be extended with repeated contaminations [39]. The riverine environment is, therefore, a choice setting for leptospiral transmission. The irrigated area also showed high exposure to leptospires and a high number of seroconverting animals. Similar contamination of soils in these areas from infected animals is likely aided by moisture from irrigation canals. A larger rodent population in these areas due to field crops and vegetation could also assist in maintaining the bacteria in the environment, leading to better transmission.

Several studies have estimated the seroprevalence of *Brucella* spp. in sheep and goats in Kenya. Seroprevalence estimates in our study were lower than what others have described in ASAL areas [6,40]. We also did not observe any seroconversions to *Brucella* spp., unlike other ASAL areas in Kenya [41].

Brucellosis features prominently in Kenya’s prioritization of transboundary animal diseases [42] and zoonotic human diseases [4]. It is also the most studied of the three zoonoses described here but was the least prevalent across all study sites. This finding supports the growing consensus that current surveillance systems are not adequate for estimating the zoonotic disease burden in animals and humans [43]. Future studies that investigate multiple diseases in various livestock species would be valuable in estimating the burden of disease attributable to specific causative agents, thereby enabling more representative disease prioritization.

We also observed no particular seasonality of seroconversions for *C. burnetii* in our study. This is contrary to findings in France which showed that dry and windy conditions might enhance transmission [44]. Seroconversions to leptospires at the end of the study were slightly more than at the beginning. We also observed slightly more seroconversions around the two wet seasons than in the dry season, even though the seroconversions observed in our study were too few to make a conclusive statement. The transmission of leptospires can be enhanced in rainy seasons [13]. Since our study period was a little under one year, we cannot draw strong conclusions on the seasonality of transmission of these pathogens. Longitudinal studies that investigate transmission over several years would, therefore, greatly help understand seasonal variations. However, it was apparent that seroconversions for both pathogens occurred throughout the study period. Therefore, continuous surveillance should be established for both pathogens regardless of weather, especially since weather patterns in the region are erratic and results based on weather patterns may not necessarily reflect the present time.

### 4.2. Co-Exposures

Our study demonstrated that for the two most prevalent exposures (*Leptospira* spp. and *C. burnetii*), animals that were exposed to one had at least 2.5 times higher odds of being exposed to the other. Caution must be taken when interpreting these findings as we used seroprevalence estimates. As such, we cannot establish which infection happened before the other, a weakness that could have been answered by using seroconversion data instead. However, due to the few data points per primary sampling unit, we did not think this was prudent. Additionally, unmeasured confounders such as age may have biased the result. Older animals are indeed more likely to be exposed to both pathogens. Similarly, unmeasured village-level characteristics may have also confounded the results, as five of the twelve animals exposed to both *C. burnetii* and *Leptospira* spp. were from Village One. However, there was no statistical significance associated with co-exposure in any village.

The high co-occurrence of seroconversions to more than one of the pathogens of interest is indicative of failed measures that can lower the zoonotic disease burden in the area. These measures could include good husbandry practices and vaccinations [45]. Co-occurring infections can place extra demand on the host’s immune system and may make animals susceptible to more serious disease outcomes [45]. Integrated surveillance systems that make use of sampling sentinel animals, households, and slaughterhouses, along with public engagement, may help inform zoonotic disease control measures in the area. Other sources of data, such as shops that sell veterinary medicines and commercial livestock farms, may also provide relevant surveillance data [46,47]. Timely reporting of sporadic abortions in livestock could contribute to syndromic surveillance systems, especially when linked to human health reporting systems, to enable early warning of potential outbreaks of zoonoses [48].

### 4.3. Serological Incidence Rates

Few studies have estimated incidence rates of the target organisms in animals for us to compare. However, higher estimates for *C. burnetii* have been reported in goats in Australia [49]. We could not find incidence rate estimates for leptospires in animal hosts. The estimates for *Brucella* spp. were much lower than what has been observed in other parts of the country closer to wildlife (1.3 cases in goats and 0.6 cases in sheep per 100 animal-months at risk) [41].

Low annual incidence rate estimates of 2.9 cases per 100,000 population have been reported for *Leptospira* spp. in humans in Kenya [50]. Annual incidence rates of 230 brucellosis cases per 100,000 population were reported in the country in 2012 [51]. However, these national human brucellosis estimates were likely overestimated due to poor test specificity and lack of confirmatory testing [51]. Annual incidence rates of 84 cases per 100,000 population have also recently been reported in humans in Kajiado County of Kenya [52]. The exceedingly high incidence rates for *C. burnetii* and *Leptospira* spp. in animal hosts underpin the public health risk in the study area and the need for disease control in animal sources to prevent human exposure and improve animal productivity.

### 4.4. Leptospiral Serovars

Only a few studies in Kenya have used MAT, despite it being the serological gold standard test for diagnosing leptospirosis. This could be because of the limited diagnostic capacity in the country, as the test is laborious and requires the maintenance of a panel of live bacterial cultures. However, the technique was used more regularly in earlier studies in the country before the advent of commercial ELISA tests, which are now commonplace.

Various serovars have been identified by MAT in sheep and goats in Kenya, with the most frequent ones being in serogroups Hebdomadis, Icterohaemorrhagiae, Sejroe, and Autumnalis [36,37,53,54]. Our study identified reference strain Mus 127 of the serovar Ballum, serogroup Ballum, as the most common. The local strain Njenga of the serovar Kenya (serogroup Ballum) was first isolated in the country in the 1980s and is likely an important locally circulating strain [55]. Other locally circulating strains such as Kibos (serovar Nyanza) [56] and Musa (serovar Ramisi) [57] of the serogroups Sejroe and Australis, respectively, have also been isolated in Kenya. The local strain RM1 (serovar Sokoine) of the serogroup Icterohaemorrhagiae was also first described in cattle in neighboring Tanzania and could have relevance in the study area [58]. These serovars were also observed in our study. The identification of serogroup Ballum, which is typically not maintained by small ruminants, also suggests that maintenance could be through other hosts, such as synanthropic rodents [13].

When we evaluated the serovars for the few animals that seroconverted, we observed variation between serovars detected in the first and last sample collection. MAT results can be confounded by cross-reaction between serovars belonging to different serogroups, especially in the acute phase of infection [13]. In addition, paradoxical reactions, which refer to higher titers being initially observed in a serogroup unrelated to the infective one, have also been documented [13]. Cross-reactivity or paradoxical reactions could have been responsible for the change in agglutinating serovars seen in the study animals. Therefore, paired sampling could add value in confirming infective serovars. Since seroconversion also takes a longer time, increasing the intervals between the paired samples or following up for longer periods and sampling repeatedly, as was done in our study, can also be beneficial. As immunity to leptospires is relatively serovar-specific, knowledge of circulating serovars is key in informing vaccine development because homologous or antigenically similar serovars should be included [13].

### 4.5. Real-Time PCR

We observed both *B. abortus* and *B. melitensis* in the small ruminants, further confirming that mixed livestock farming, which is practiced in the study area, is a risk factor for the spread of animal brucellosis in Kenya [59,60]. We further noted animals testing positive by PCR for 7 to 10 months. Sheep and goats can actively shed brucellae and have blood testing PCR-positive for long periods after initial infection [61,62]. The extended period animals can shed the bacteria enables them to remain infective for a long time.

Similarly, we observed one sheep that tested positive for *C. burnetii* by PCR once and later seroconverted. Sheep can shed *C. burnetii*, mainly in feces and vaginal mucus, for long periods after initial exposure, resulting in disease outbreaks in humans [63]. It is possible that the animal only had bacteria below the limit of PCR detection in the other time points or testing positive via samples other than blood. Since the ewe was reproductively active and from a large herd, she could still have continued transmitting the bacteria to other members of the household, posing a serious public health risk. Obtaining other samples such as milk, vaginal swabs, and feces could have shed more light on the risk of disease exposure. However, this was not done due to limited resources.

Our study did not detect pathogenic leptospires by PCR, despite having several animals seroconverting and testing positive by MAT. The acute stage of leptospirosis is often characterized by bacteremia, and infected hosts are likely to be PCR-positive via blood [13]. However, as the disease progresses to the convalescent stage, antibody titers rise and leptospires are excreted in urine [13]. Testing of urine samples by PCR could therefore have given a better view of the exposure status of the study animals. However, this was also not conducted due to limited resources.

## 5. Conclusions

The spread of *C. burnetii* could be enhanced in irrigated and pastoral settings when compared to riverine ones. Therefore, there is a need to invest in the vaccination of herds against coxiellosis, especially with Kenya’s current efforts to promote irrigation agriculture. There is also a need to control environmental transmission with measures such as rodent control and using properly composted manure.

Additionally, brucellosis, which is a frequently studied disease, was less frequent in the study area. The less studied zoonoses (coxiellosis and leptospirosis) were much more frequent, highlighting the need for systematic prioritization of animal diseases. This will enable the use of limited resources to address zoonotic diseases according to their importance in the local context.

Since animals that were exposed to one zoonosis were also more likely to be exposed to a second one, disease surveillance and control measures can be integrated to boost production and improve livelihoods. Greater investment is needed to foster better coordination of human, animal, and environmental zoonotic disease control measures in the region. This is vital in Kenya’s ASAL regions, which though historically marginalized, hold immense potential for livestock production for both the local and export markets.

## Figures and Tables

**Table 1:** A summary of the primers and probes used to detect *Brucella* spp., pathogenic leptospires, and *C. burnetii*.

Organism (PCR Target)	Primer/Probe	Sequence and Modifications (5′ → 3′)	Reference
*Brucella* spp. (IS*711* element)	Forward	GCTTGAAGCTTGCGGACAGT	[21]
	Reverse	GGCCTACCGCTGCGAAT	
	Probe	FAM-AAGCCAACACCCGGCCATTATGGT-BHQ1	
*Brucella* spp. (*bcsp31* gene)	Forward	GCTCGGTTGCCAATATCAATGC	[22]
	Reverse	GGGTAAAGCGTCGCCAGAAG	
	Probe	FAM-AAATCTTCCACCTTGCCCTTGCCATCA-BHQ1	
*B. abortus* (IS*711* element downstream of *alkB* gene)	Forward	GCGGCTTTTCTATCACGGTATTC	[22]
	Reverse	CATGCGCTATGATCTGGTTACG	
	Probe	HEX-CGCTCATGCTCGCCAGACTTCAATG-BHQ2	
*B. melitensis* (IS*711* element downstream of BMEI1162)	Forward	AACAAGCGGCACCCCTAAAA	[22]
	Reverse	CATGCGCTATGATCTGGTTACG	
	Probe	TxRd-CAGGAGTGTTTCGGCTCAGAATAATCCACA-BHQ2	
Pathogenic leptospires (*lipL32* gene)	Forward	AAGCATTACCGCTTGTGGTG	[24]
	Reverse	GAACTCCCATTTCAGCGAT	[25]
	Probe	FAM-AAAGCCAGGACAAGCGCCG-BHQ1	[24]
*Coxiella burnetii* (IS*1111* element)	Forward	GTCTTAAGGTGGGCTGCGTG	[26]
	Reverse	CCCCGAATCTCATTGATCAGC	
	Probe	FAM-AGCGAACCATTGGTATCGGACGTTTATGG-TAMRA	

**Table 2:** The distribution of animals seropositive for *C. burnetii*, *Leptospira*, and *Brucella* with animal and household characteristics. The study period was between September 2014 and June 2015.

Category	Variable	First Sampling Time Points			Seroconversions		
		Positives	Total	Percentage (%)	Positives	Total ^†^	Percentage (%)
*Coxiella burnetii*							
Total		66	316	20.9	10	218	4.6
Age	Young	9	110	8.2	4	86	4.7
	Adult	57	207	27.5	6	132	4.5
Sex	Female	61	252	24.2	9	171	5.3
	Male	5	64	7.8	1	47	2.1
Species	Goat	58	228	25.4	8	148	5.4
	Sheep	8	88	9.1	2	70	2.9
Herd size	<13	25	142	17.6	2	103	1.9
	13–35	13	98	13.3	1	72	1.4
	>35	28	76	36.8	7	43	16.3
Reproductive status	Active	64	260	24.6	10	172	5.8
	Inactive	2	56	3.6	0	46	0.0
*Leptospira* spp.							
Total ^‡^		48	313	15.3	27	226	11.9
Age	Young	8	108	7.4	6	85	7.1
	Adult	40	205	19.5	21	141	14.9
Sex	Female	44	252	17.5	22	180	12.2
	Male	4	62	6.5	5	46	10.9
Species	Goat	44	227	19.4	19	154	12.3
	Sheep	4	86	4.7	8	72	11.1
Herd size	<20	31	187	16.6	13	137	9.5
	20–40	9	69	13.0	10	45	22.2
	>40	8	57	14.0	4	44	9.1
Reproductive status	Active	43	257	16.7	27	183	14.8
	Inactive	5	56	8.9	0	43	0.0
*Brucella* spp.							
Total		4	316	1.3	0	267	0.0
Age	Young	1	110	0.9	-	-	-
	Adult	3	206	1.5	-	-	-
Sex	Female	3	252	1.2	-	-	-
	Male	1	64	1.6	-	-	-
Species	Goat	3	228	1.3	-	-	-
	Sheep	1	88	1.1	-	-	-
Herd size	<13	1	142	0.7	-	-	-
	13–35	2	98	2.0	-	-	-
	>35	1	76	1.3	-	-	-
Reproductive status	Active	4	260	1.5	-	-	-
	Inactive	0	56	0.0	-	-	-

^†^ Totals comprised animals that were seronegative at the time of first sampling, and animals sampled only once were excluded. Therefore, totals for each pathogen vary. ^‡^ Three animals were removed from the analyses because they had doubtful results and were lost to follow-up.

**Table 3:** The distribution of seroprevalence estimates for the three bacterial zoonoses. Seroprevalence was calculated using the first sampling time point of all animals. When the weighted estimates could not be reliably determined, the cells are left blank and only the unadjusted numbers are given.

Weighted Seroprevalence					
Category	Variable	Positives	Total	% (95% CI)	SE
*Coxiella burnetii*					
Total		66	316	34.6 (24.3–47.0)	4.7
Site	Irrigated	34	139	24.1 (7.9–54.0)	9.3
	Pastoral	25	69	38.7 (17.4–65.0)	8.2
	Riverine	7	108	4.4 (1.87–10.0)	1.7
*Leptospira* spp.					
Total ^†^		48	313	15.3 (11.6–20.0)	1.7
Site	Irrigated	15	138	17.2 (6.8–37.0)	5.8
	Pastoral	8	68	13.9 (5.3–32.0)	4.0
	Riverine	25	107	26.9 (15.5–42.0)	6.2
*Brucella* spp.					
Total		4	316	-	-
Site	Irrigated	3	139	-	-
	Pastoral	0	69	-	-
	Riverine	1	108	-	-

CI: Confidence interval; SE: Standard Error. ^†^ Three animals were removed from the analyses because they had doubtful results and were lost to follow-up.

**Table 4:** Results of the survey-weighted univariable logistic regression models of seroprevalence for the different sampling sites. Due to the few positives, data on *Brucella* spp. was not analyzed. Models were built using a causal diagram-based approach to determine total effects. Therefore, all intervening variables were excluded from the analyses.

		Odds Ratio				
Agent	Variable	Estimate	Lower 95% CI	Upper 95% CI	SE	*p*-Value
*Coxiella burnetii*	Riverine	Ref.				
	Irrigated	6.83	2.58	18.06	0.50	0.01
	Pastoral	13.61	13.61	13.61	0.00	0.00
Observations = 316; Pseudo-R² (McFadden) = 0.05						
*Leptospira* spp.	Irrigated	Ref.				
	Pastoral	0.78	0.36	1.70	0.40	0.56
	Riverine	1.77	0.81	3.88	0.40	0.21
Observations = 313 ^†^; Pseudo-R^2^ (McFadden) = 0.01						

CI: Confidence interval; SE: Standard error; Ref.: Reference category. ^†^ Three animals were removed from the analyses because they had doubtful results and were lost to follow-up.

**Table 5:** Results of the final survey-weighted multivariable logistic regression models for *Coxiella burnetii* and *Leptospira* spp. Models represent the direct effects of variables on seropositivity and therefore include intervening variables as determined by the causal diagram.

		Odds Ratio					
Agent	Variable	Category	Estimate	Lower 95% CI	Upper 95% CI	SE	*p*-Value
*Coxiella burnetii*	Site						
		Riverine	Ref.				
		Irrigated	6.13	2.46	15.27	0.47	0.06
		Pastoral	13.61	4.99	34.70	0.49	0.03
	Age						
		Young	Ref.				
		Adult	17.88	9.79	32.66	0.31	0.01
	Herd size						
		<13	Ref.				
		13–35	0.45	0.13	1.49	0.61	0.32
		>35	1.33	0.29	6.01	0.77	0.75
Observations = 316; Pseudo-R^2^ (McFadden) = 0.26							
*Leptospira* spp.	Site						
		Irrigated	Ref.				
		Pastoral	0.59	0.24	1.48	0.47	0.46
		Riverine	1.89	0.74	4.84	0.48	0.41
	Age						
		Adult	Ref.				
		Young	0.10	0.01	0.71	1.01	0.26
	Sex						
		Female	Ref.				
		Male	0.12	0.01	1.62	1.33	0.36
	Species						
		Goat	Ref.				
		Sheep	0.05	0.00	0.69	1.36	0.27
	Reproductive status						
		Inactive	Ref.				
		Active	0.08	0.00	1.75	1.55	0.36
Observations = 313 ^†^; Pseudo-R^2^ (McFadden) = 0.17							

CI: Confidence interval; SE: Standard error; Ref.: Reference category. ^†^ Three animals were removed from the analyses because they had doubtful results and were lost to follow-up.

**Table 6:** Summary of the Cq values and serological results of the four animals that were PCR-positive for *Brucella* spp.

Animal ID	Sampling Date	Pan-*Brucella* qPCR		Species-Specific qPCR		Serological Assays		
		IS*711*	*bscp31*	*B. melitensis*	*B. abortus*	CFT	ELISA	RBT
48	September 2014	-	-	-	-	positive	negative	negative
48	November 2014	39.8	-	-	-	negative	negative	negative
48	December 2014	37.7	-	-	-	negative	negative	negative
48	January 2015	37.9	39.1	-	-	negative	negative	negative
48	March 2015	37	-	-	-	negative	negative	negative
48	June 2015	38.7	-	-	-	negative	negative	negative
102	September 2014	35.2	-	40	38.6	positive	positive	negative
115	September 2014	-	-	-	-	positive	negative	negative
115	November 2014	-	38.8	-	-	negative	negative	negative
115	December 2014	37.4	-	-	-	negative	negative	negative
115	February 2015	-	39	-	-	negative	negative	negative
115	June 2015	38.1	38.9	-	-	negative	negative	negative
370	August 2014	39.8	-	-	-	positive	negative	negative
370	October 2014	39.3	-	-	-	positive	negative	negative
370	November 2014	-	-	-	-	positive	negative	negative
370	January 2015	36.8	-	-	-	positive	negative	negative
370	March 2015	36.9	-	-	39.1	positive	negative	negative
370	June 2015	37.3	-	-	-	positive	negative	negative

CFT: Complement fixation test; qPCR: Real-time PCR; ELISA: Enzyme-linked immunosorbent assay; RBT: Rose Bengal test.

## Data Availability

Data will be made available from the authors upon request.
